# The epidemiology and risk factors of anal and oropharyngeal human papillomavirus (HPV) in males living with human immunodeficiency virus (HIV): A protocol for systematic review and meta-analysis

**DOI:** 10.1371/journal.pone.0320899

**Published:** 2025-05-07

**Authors:** Mengni Zhang, Yajing Huang, Shipeng Zhang, Shunlin Peng

**Affiliations:** Department of Otolaryngology, Hospital of Chengdu University of Traditional Chinese Medicine, Chengdu, China; Kampala International University - Western Campus, UGANDA

## Abstract

**Background:**

People living with human immunodeficiency virus (HIV) are more likely to be infected with human papillomavirus (HPV), which can lead to anal and oropharyngeal cancer in males, increasing the complexity of the disease.

**Objective:**

The aim of this study is to learn more about epidemiological data and factors influencing anal and oropharyngeal HPV infections in males with HIV through a systematic literature review in order to provide evidence for future interventions.

**Methods:**

The PubMed, Embase, Web of Science, Cochrane Library, PsycINFO, and ClinicalTrials.gov databases will be searched from build to June 2024. Two researchers will independently carry out study selection, data extraction and risk of bias assessment. STATA 16.0 will be used to conduct meta-analysis. The estimated pooled prevalence of anal and oropharyngeal HPV (any HPV, high-risk HPV, low-risk HPV, type-specific HPV, and multiple HPV) in males living with HIV will be presented as percentage prevalence (p * 100%) with corresponding 95% confidence intervals (CI) by DerSimonian-Laird random-effect meta-analyses. Subgroup analysis will carried out by geographic region, income level, race, age, publication year, HPV genotyping, and risk factors (e.g., sexual behavior patterns). Risk factors and interventions-related dichotomous variables or continuous variables will be summarized as odds ratios (OR) and standardized mean differences, and reported through forest plots. The OR (greater than 1) shows an association (correlated). Heterogeneity will be assessed using the I^2^. Funnel plots, Begg and Egger tests will be applied to detect potential publication bias.

**PROSPERO registration number:**

CRD42024579641.

## 1. Introduction

People living with human immunodeficiency virus (HIV) are more susceptible to several cancers owing to their behavioral and biological characteristics, immunodeficiency, and possibly chronic inflammation. Impairment of immunity by HIV reduces the body’s resistance to cancer-causing viruses [1]. Human papillomavirus (HPV) is the most prevalent sexually transmitted infection (STI) worldwide [2]. Infectious HPV viruses attach to basal skin stem cells through subtle destruction of epithelial cells. Although most HPV infections resolve spontaneously, several diseases that weaken the immune system contribute to the persistence of the infection [3]. High-risk HPV types are thought to cause 7–8% of human malignancies, including 96% of cervical cancers, 93% of anal cancers, 64% of vaginal cancers, 51% of vulvar cancers, 36% of penile cancers, and 63% of oropharyngeal cancers (OPC) [4,5].

Current data suggest that HIV-infected individuals have a higher prevalence of HPV infections, are more likely to carry multiple HPV types, and have higher HPV persistence rates than HIV-negative individuals [6]. HIV-infected people are more likely to develop precancerous lesions that resolve more slowly [7]. Co-infection with HIV and HPV increases the risk of a variety of diseases [8]. A weakened immune system can trigger this increased risk because when a person’s CD4 levels fall, the likelihood of generalized HPV infection of the anus, cervix, oropharynx, penis, vagina, and vulva increases [9]. The molecular interactions between the two viruses are not fully understood. The HIV tat protein transactivates the HPV long control region and increases the expression of the oncogenes E6 and E7. The HIV tat protein promotes cell cycle progression and reduces the expression of cell cycle inhibitors. HIV infection induces a state of immunosuppression by decreasing the level of CD4+ lymphocytes, impairing dendritic cell activation and CD8+ lymphocyte activity. And HIV activity on CD4+ and CD8+ lymphocytes may affect HPV clearance by epithelial cells and promote cell cycle dysregulation [8].

Anal cancer is uncommon in the general population, with a global incidence of only 1 in 100,000. However, it has been reported that the incidence and mortality of anal cancer have increased over the past four decades in various countries, particularly in the more industrialized regions [10]. Anal squamous cell carcinoma is now one of the fastest growing causes of cancer incidence and mortality in the United States [11]. HIV-infected people have a significantly higher risk of developing anal cancer than HIV-negative people [12]. Gopalani et al. analyzed data from the US Cancer Statistics and found that the incidence of HPV-associated squamous cell carcinomas of the anus was unequal in terms of geographic and county-level economic characteristics [13]. Further epidemiological data from the United States showed that neighborhood socioeconomic status (NSEs), ethnicity, and their interactions were strongly linked to the incidence and survival trends of HPV-related anal cancer [14]. In addition, bad sexual habits, including early age of sexual debut, having too many sexual partners, and not using condoms, have been shown to be risk factors for anal cancer.

OPC, particularly oropharyngeal squamous cell carcinoma (OPSCC) risk, has historically been associated with high tobacco and alcohol use. However, oncogenic HPV infection has recently driven the increase in OPC [15]. The crypts and irregular surfaces of the tonsils and lymphoid tissue in the base of the tongue are generally believed to provide a supportive environment for HPV infection to persist [16]. The incidence of HPV-related OPSCC in men has surpassed the incidence of cervical cancer in women in the United States and the United Kingdom, with a similar trend in continental Europe [17]. Sexual activity may be the reason why OPSCC is more common in developed industrialized countries and Western societies. In the United States, the overall annual incidence rate is around 5 cases per 100,000 people, with the majority of new cases among white men aged 65 and younger. Whiteness, relative youth, and relatively high socio-economic status are associated with increased incidence [18].

Over the last decade, it has become widely recognized that there are differences in immune response between males and females [19]. The reasons for the higher rates of oral HPV infections and HPV-positive oropharyngeal cancer in males than in females are unclear. Possible reasons include a higher number of lifelong sexual partners in males, lower seroconversion rates after HPV infection in males (hormonal differences), and a significantly stronger association between male sexual behavior and oral HPV infection than in females [20,21]. HIV-infected men who have sex with men (MSM) have the highest rates of anal infections with high-risk human tumor viruses (HR-HPVs) and the highest incidence of anal cancer [22]. Data suggest that the incidence of anal cancer in the general population is 1–2/100,000, while the incidence of anal cancer in HIV-infected MSM is 131/100,000, regardless of whether they are receiving antiretroviral therapy or not [23]. It is known that HIV occurs primarily in backward regions such as Africa. In high-income countries, the majority of the HIV burden is concentrated in the MSM population. The burden of HPV-related diseases is disproportionately concentrated in low- and middle-income countries [24]. The prevalence of anal and oral HPV infection in males with HIV may varies by region. In a study conducted in the Czech Republic involving 205 MSM, the prevalence of anal HPV infection was 96.8% and oral HPV infection was 23.6% [25]. In a survey conducted in France including 421 MSM, multiple HPV types were detected in 70% of anal samples and a single HPV type was detected in 91% of oropharyngeal samples [26].

Since the first HPV vaccine was approved in 2006, HPV vaccination programs have been implemented in approximately 100 countries [7]. A recent systematic review found that studies of anogenital HPV cancer in women living with HIV in Europe focused primarily on cervical cancer prevention, with only 4.4% of studies focusing on anal cancer [27]. The effectiveness of HPV vaccines in preventing and treating cervical cancer in females has been increasingly demonstrated. Seroconversion rates and vaccine-related geometric mean titers were lower in people living with HIV than in immunocompetent participants, particularly in patients with CD4 cell counts below 200 cells/mm^3^ and detectable viral loads [6]. There is some controversy as to whether or not HIV infection negatively impacts vaccine-mediated protection. Data presented in Rossotti’s study suggested that while people living with HIV had a higher prevalence of oral HPV infection, HIV had no effect on viral clearance or infection following vaccination [28]. Randomized trials conducted by Wilkin found no benefit for HPV vaccination to prevent new anal HPV infections. It may be beneficial in preventing oral HPV infection [29].

To the best of our knowledge, there is no systematic analysis of epidemiological data on HPV infections in the anus and oral cavity in males living with HIV in the literature. In this systematic review, we will summarize the available data and describe the epidemiological data on the prevalence, incidence, clearance and persistence of anal and oropharyngeal HPV in males living with HIV, taking into account the various influencing factors associated with the infection. The goal is to identify modifiable risk factors and interventions to maximize health benefits. These reviews and meta-analyses will synthesize evidence from all of these populations to provide recommendations for public health resource allocation.

## 2. Methods

### 2.1. Study registration

This study was registered with PROSPERO (CRD42024579641.) on August 27, 2024. This protocol conforms to the Preferred Reporting Items for Systematic Review and Meta-Analysis Protocols (PRISMA-P) guidelines. We will report on the results of this systematic evaluation according to PRISMA. The systematic evaluation will be obtained from the published literature and does not require ethical approval. The findings will be synthesized and analyzed for publication in a peer-reviewed journal.

### 2.2. Patient and public involvement

Patients and/or the public will not involved in the design, conduct, reporting, or dissemination plans of this research.

### 2.3. Inclusion criteria and exclusion criteria

We will include observational and interventional studies with primary data using cross-sectional, cohort (prospective and retrospective), case-control designs rand randomized controlled trials (RCTs). Epidemiological studies report prevalence and risk factor analyzes of anal (anal swab) or oropharyngeal (oral rinse and saliva samples) HPV will be included in the meta-analysis. Studies on the incidence, clearance, persistence of HPV will be excluded from the meta-analysis, but we will provide a comprehensive narrative of these data. Intervention studies will report the effectiveness of interventions to prevent or treat anal and oropharyngeal HPV infections in males living with HIV.

Risk factors of concern include unprotected sex, multiple sexual partners, oral sex, intimate kissing, tonsillectomy, advanced age, smoking, alcohol consumption, low CD4+ T cell counts, high viral loads, and a history of other sexually transmitted diseases. Interventions of interest include HPV vaccination, circumcision, and other interventions that may occur.

Inclusion criteria: (1) The subjects included in the study will be male patients with a clear diagnosis of HIV infection. (2) Anal and oropharyngeal HPV prevalence assessment by polymerase chain reaction (PCR), hybridization capture, or other well-described genotyping method. (3) Outcome indicators for the intervention include anal and oropharyngeal HPV prevalence data, serum immunogenicity data (seroconversion rate and geometric mean titer (GMT)).

Exclusion criteria: (1) Duplicate studies or study data from the same area (2) Systematic review (3) Case report (4) Animal study (5) Replicated study (6) Non-English study (7) Study with incomplete data (8) Lower quality studies (9) Studies concentrated on malignant lesions of anus and oropharynx

### 2.4. Information sources and search strategy

This investigation will carry out an extensive and comprehensive literature search in the databases (PubMed, Embase, Web of Science, Cochrane Library, PsycINFO, and ClinicalTrials.gov). All databases will be searched from inception to June 2024. The following keywords and Medical Subject Heading will be used to select relevant studies: “HIV”, ”Human immunodeficiency virus”, ”Human immunodeficiency viruses”, ”AIDS Virus”, ”AIDS Viruses”, ”Acquired Immune Deficiency Syndrome Virus”, ”Acquired Immunodeficiency Syndrome Virus”, ”Human Papillomavirus Viruses”, ”HPV”, ”Human Papillomavirus Virus”, ”Human Papilloma virus”, ”Human Papilloma Viruses”. PubMed search strategy is (“HIV” OR “Human immunodeficiency virus” OR “Human immunodeficiency viruses” OR “AIDS Virus” OR “AIDS Viruses” OR “Acquired Immune Deficiency Syndrome Virus” OR “Acquired Immunodeficiency Syndrome Virus”) AND (“Human Papillomavirus Viruses” OR “HPV” OR “Human Papillomavirus Virus” OR “Human Papilloma virus” OR “Human Papilloma Viruses”) AND (“Men” OR “Boys” OR “Males”) AND (“Anal” OR “Anus” OR “Archos” OR “Oropharyngeal” OR “Oral” OR “Oropharynx” OR “Mouth”). In addition, all reference lists will be searched to identify any missing potential studies that may be eligible.

### 2.5. Study selection and data extraction

**Fig 1:**
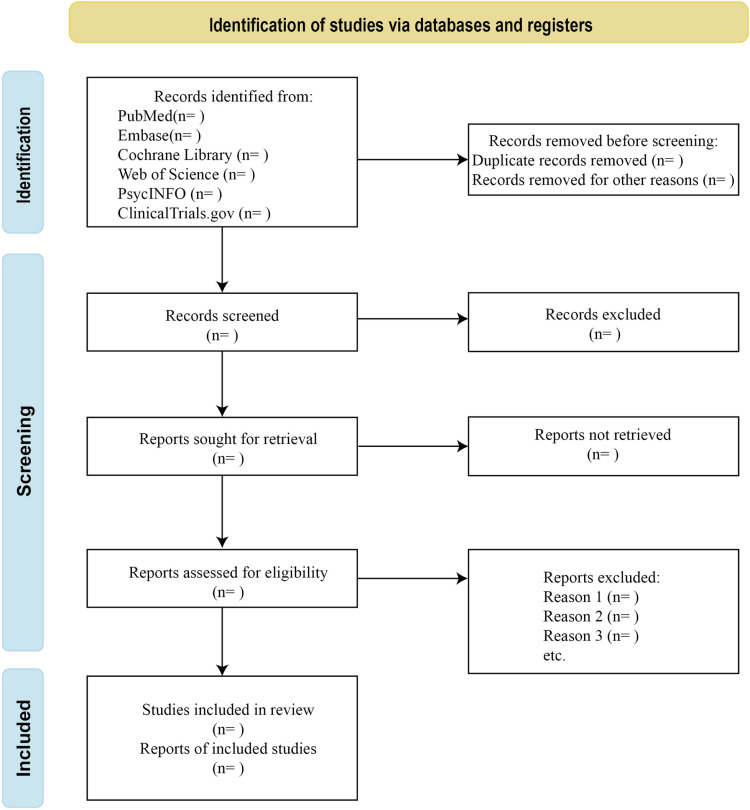
Study process.

Two researchers (M-NZ and S-PZ) will independently conduct a literature selection based on inclusion and exclusion criteria. First, titles and abstracts will be reviewed to list articles that meet the inclusion criteria, and the full text will be collected for further assessment for inclusion/exclusion by professional literature management software EndNote X9. Reasons for exclusion will be documented. Fig 1 shows the study process. A pre-designed data extraction form will be used to extract relevant research data. Any disagreements that arise during the extraction process will be resolved through discussion and consultation with the third reviewer. The following data will be listed: first author, year of publication, study design, location (country, region, or city), study period, duration of follow-up, inclusion criteria, exclusion criteria, total sample size, characteristics (age, ethnicity, etc.), sexual behavior pattern, age of HIV infection, duration of treatment, CD4+ cell count, HIV viral load, specimen collection, methods of HPV testing and genotyping, number of HPV positive and negative samples.

We will evaluate the following outcomes: anal and oropharyngeal HPV prevalence, high-risk HPV (16/18/31/33/35/39/45/51/52/56/58/59/68) prevalence, low-risk HPV (6/11/40/42/43/44/54/61/72/81) prevalence, type-specific HPV prevalence and multiple HPV prevalence; crude and adjusted association metrics (ratio, relative risk, risk ratio, etc.) between prevalence and associated exposures, and confounding and modifying factors.

### 2.6. Assessment of study quality and publication bias

Two researchers (M-NZ and S-PZ) will independently investigate the methodological quality of cohort and cross-sectional studies using the National Institutes of Health quality assessment tool. By examining 14 individual points, each study will be assigned an overall quality rating of good, fair or poor. The Cochrane risk-of-bias tool for randomized trials (RoB2), a well-validated standardized approach for assessing the risk of bias in RCTs will be used to assess the risk of bias in RCTs. The tool consists of seven domains: (1) random sequence generation, (2) allocation concealment, (3) blinding of participants and personnel, (4) blinding of outcome assessments, (5) incomplete outcome data, (6) selective reporting, and (7) other biases. The risk of bias for each area will be assigned as ‘low’, ‘high’, or ‘unclear’. In case of any disagreement, further investigation and final decision will be made by a third researcher (S-LP).

### 2.7. Statistical analysis

#### 2.7.1. Prevalence meta-analysis.

The data will be entered using Microsoft Excel. The prevalence of HPV infection, i.e., at least one HPV infection, although participants can have multiple HPV infections, will be counted as one event. We will estimate the prevalence of HPV infection as the number of individuals carrying any HPV genotype divided by the total number of individuals tested for HPV. The prevalence of HR-HPV infection will be calculated as the number of participants carrying any of the 13 genotypes divided by the total number of participants tested for HPV. The prevalence of LR-HPV infection will be calculated in the same way. For the prevalence of specific HPV types, the sample size varies depending on the HPV type, as we will only include studies that tested specific HPV types. To conduct a meta-analysis of the prevalence data, we will first transform the prevalence from each study using the Freeman-Tukey double arcsine transformation. The arcsine transformations are necessary to stabilize the variance of simple proportions. And DerSimonian-Laird random-effect meta-analyses will be used to calculate pooled prevalence performed by STATA V.16.0 (Stata Corporation, College Station, Texas, USA) [30–32].

### 2.8. Risk factors meta-analysis

Meta-analyses will be performed when there are three or more comparisons regarding risk factors and interventions, and the study findings will be synthesized narratively when there are fewer than three comparisons. The binary results will be analyzed by STATA V.16.0 to obtain a pooled summary odds ratios (OR), and each result will be presented with its 95% confidence interval (CI). Effect measures reported as hazards ratios, risk ratios or relative risks will be transformed into OR using standard methods. Seroconversion will indicate the concentration of HPV-specific antibodies above a threshold, and the seroconversion rate will be defined as the proportion of seropositive participants. For geometric mean titer (GMT), anti-ln will be carried out to ensure normality and the results will be shown as standardized mean difference (SMD) along with 95% CI. Heterogeneity between studies will be estimated by I^2^ (< 25%, no heterogeneity; 25%-50%, moderate heterogeneity; > 50%, strong heterogeneity). If no heterogeneity exists, a fixed-effects model will be conducted, and if heterogeneity exists (*p* < 0.1, I^2^ > 50%), a random-effects model will be conducted.

### 2.9. Dealing with missing data

If certain information is missing from the included studies, attempts will be made to contact the authors by email. If missing data are still not available, these studies will be excluded from the analysis.

### 2.10. Assessment of publication bias

If more than 10 studies are ultimately included, funnel plots and Begg and Egger tests will be applied to detect potential publication bias. An asymmetrical funnel plot represents publication bias, and p < 0.05 indicates statistically significant publication bias by Begg and Egger tests.

### 2.11. Subgroup analysis

When there is significant heterogeneity, we will conduct subgroup analyzes based on geographical region, income level as defined by the Gross Nation Income (GNI) World Bank Classification (lower-middle income, upper-middle income, high income), race, age, publication year, HPV genotyping, and risk factors (e.g., sexual behavior patterns) to investigate possible causes of heterogeneity.

### 2.12. Sensitivity analysis

We will conduct sensitivity analyzes to test the robustness of the effect estimates generated by the meta-analysis. Sensitivity analyses will be used to assess the impact of individual outliers on the overall estimate, and will also be used to estimate the effect of missing data and unpublished studies.

### 2.13. Quality of evidence

Two researchers (M-NZ and S-PZ) will estimate the quality of evidence according to the Grading of Recommendations Assessment, Development and Evaluation (GRADE) system, and the outputs will be expressed as ‘moderate’, ‘low’ or ‘critically low’. The third researcher will cross-check the results, and any divergences that arise will be resolved by the third assessor (S-LP).

## 3. Discussion

Coinfection with HIV and HPV significantly affects the survival and recovery of male patients. Despite high survival rates, long-term toxicity and poor functional outcomes remain a concern for cancer survivors because the oropharynx is essential for important daily functions such as speech, swallowing, and airway patency [33]. In addition, people living with HIV may suffer greater harm. One study conducted in Washington, D.C., showed that HIV-infected individuals survived less than a year after being diagnosed with oropharyngeal cancer [9]. HPV-positive oral cancer has been shown to be an important contributor to morbidity and mortality in human HIV-infected individuals [1]. A recent meta-analysis showed that HPV16 E6 seropositive individuals had a 3.6-fold increased risk of high-grade intraepithelial squamous cell lesions and a 26.1-fold increased risk of anal cancer [34]. People living with HIV have an increased incidence of anal cancer, particularly MSM, which may be 40–60 times higher than in the general population [35].

There is a need to understand the prevalence, incidence, clearance, persistence and factors affecting HPV in males living with HIV in different regions. There is currently a relatively large amount of relevant literature and a rigorous synthesis of the evidence is required. Anal and oropharyngeal HPV prevalence in male HIV patients will be analyzed by geographic region, income level, race, age, publication year, HPV genotyping, and risk factors. Although pepole living with HIV are considered a high priority group for HPV vaccination, there are limited data on the long-term immunogenicity and efficacy of HPV vaccines in this population [6]. Males are also significantly less likely to get vaccinated than females: 44% in developed countries and 5% in developing countries [36]. Circumcision is believed to be effective for both HIV and HPV infections [8]. However, high-quality studies are required for systematic analysis and synthesis. Clear effects of interventions on outcomes may help control HPV transmission and cure.

We will electronically search various search engines for the first comprehensive systematic review estimating the pooled prevalence and risk factors for anal and oropharyngeal HPV in males living with HIV worldwide. We will review completed studies to identify gaps in this phase and provide some ideas for future study designs. Established clear inclusion and exclusion criteria will provide accurate data for this systematic review. The search will carry out without time or geographical restrictions. This review will result in modifiable behavioral risk factors, attitudes, and beneficial interventions. This work will provide researchers, policymakers, and public health stakeholders with some insights necessary to establish research, policy, and program priorities for HPV prevention, control, and treatment in males living with HIV.

## Strengths and limitations of this study

- This study will conduct a comprehensive search to evaluate global epidemiological data on anal and oropharyngeal HPV of males living with HIV.
- The risk factors and beneficial interventions for anal and oropharyngeal HPV of males living with HIV will be evidenced.
- Rigorous inclusion and exclusion criteria will be used in this study and robust analyses will be conducted to enhance the reliability of the results.
- The results may be affected by the number and quality of included studies, as well as by language bias.

## Supporting information

S1 Fig PRISMA-P-checklist. (DOC)
